# Supplementary material for: Comprehensive Longitudinal Linear Mixed Modeling of CTCs Illuminates the Role of Trop2, EpCAM, and CD45 in CTC Clustering and Metastasis
Source: Cancers (Basel). 2025 Aug 21;17(16):2717. doi: 10.3390/cancers17162717 (PMC12384426; doi:10.3390/cancers17162717)
Supplement: Supplementary file 1 [file cancers-17-02717-s001.zip › cancers-3803740-supplementary.pdf]

Table S1. Full and Reduced model results for cCTC analysis by receptor status and metastatic site

cCTC Longitudinal Analysis by receptor status - full model

Biomarker	Receptor Effect	Time Effect	Interaction
cCTCs	0.6591061	0.7461176	0.840692
CK	0.4685809	0.6062315	0.4074604
**EpCAM**	0.7158591	0.1999038	**0.0218357**
Clusters	0.9510659	0.028952	0.0674635
Clustersof2	0.6826174	0.0805827	0.1121893
cCTCincluster	0.4323639	0.5820074	0.5686966
**Clusters>2**	0.5347161	0.0062573	**0.0069147**
CD45 incluster	0.9598183	0.0401535	0.1091464

cCTC Longitudinal Analysis by receptor status - reduced model

Biomarker	Receptor Effect	Time Effect
cCTCs	0.6626405	0.7888826
CK	0.9279099	0.5090617
**EpCAM**	**0.0750713**	0.5069871
**Clusters**	0.0413095	**0.0948638**
Clusters of 2	0.0165396	0.2219084
cCTCincluster	0.6089088	0.7988391
**Clusters>2**	**0.0676509**	**0.0496504**
CD45 in cluster	0.5724358	0.3317939

cCTC Longitudinal Analysis by lung mets - full model

Biomarker	Lung metastasis	Time Effect	Interaction
cCTCs permL	0.189067	0.8545727	0.7152982
CK permL	0.4511593	0.1919138	0.4065616
EpCAM permL	0.277111	0.8373921	0.4649043
Clusters permL	0.2248947	0.1601939	0.8591546
Clustersof2permL	0.4846009	0.2147459	0.7757324
cCTCincluster permL	0.7143193	0.6190222	0.8204585
Clusters>2 permL	0.2505	0.0975594	0.8099052
CD45 incluster permL	0.2618324	0.0884779	0.9418482

cCTC Longitudinal Analysis by lung mets - reduced model

Biomarker	Lung metastasis	Time Effect
cCTCs permL	0.1240655	0.7676122
CK permL	0.055387	0.248619
EpCAM permL	0.7351751	0.9013928
Clusters permL	0.0935574	0.1261267
Clustersof2permL	0.1562739	0.2196801
cCTCincluster permL	0.3314729	0.6678029
**Clusters>2 permL**	0.1225333	**0.0721133**
**CD45 incluster permL**	0.1016866	**0.0776259**

cCTC Longitudinal Analysis by bone mets - full model

Biomarker	Bone metastasis	Time Effect	Interaction
cCTCs permL	0.4537911	0.5219091	0.6081255
CK permL	0.6074138	0.6430672	0.6920662
EpCAM permL	0.8281511	0.6013451	0.6301315
Clusters permL	0.4912308	0.1528036	0.8951054
Clustersof2permL	0.6176345	0.2224079	0.7702274
cCTCincluster permL	0.2492102	0.3851969	0.766914
Clusters>2 permL	0.7957817	0.1302366	0.7163378
CD45 incluster permL	0.9370934	0.0661155	0.4625753

cCTC Longitudinal Analysis by bone mets - reduced model

Biomarker	Bone Metastasis	Time Effect
cCTCs permL	0.1203738	0.6461721
CK permL	0.7442644	0.4443496
EpCAM permL	0.2891943	0.6161733
Clusters permL	0.3319319	0.1759581
Clustersof2permL	0.3154752	0.2113488
cCTCincluster permL	0.1256006	0.2219581
**Clusters>2 permL**	0.3457841	**0.0697864**
**CD45 incluster permL**	0.4165274	**0.0909111**

cCTC Longitudinal Analysis by brain mets - full model

Biomarker	Brain metastasis	Time Effect	Interaction
cCTCs permL	0.4891426	0.8125237	0.7165565
CK permL	0.7635047	0.11276	0.1055117
EpCAM permL	0.9538765	0.7597486	0.9133053
Clusters permL	0.9793359	0.1017716	0.4030987
Clustersof2permL	0.8669048	0.1582194	0.2138871
cCTCincluster permL	0.8869657	0.9178424	0.3627807
Clusters>2 permL	0.889335	0.0770817	0.6993947
CD45 incluster permL	0.669624	0.0858838	0.4493501

cCTC Longitudinal Analysis by brain mets - reduced model

Biomarker	Brain metastasis	Time Effect
cCTCs permL	0.1730142	0.9184405
CK permL	0.3169089	0.3183411
EpCAM permL	0.8362901	0.6806761
Clusters permL	0.1882687	0.4000413
Clustersof2permL	0.2557904	0.2831323
cCTCincluster permL	0.1972436	0.5785443
**Clusters>2 permL**	0.517767	**0.079834**
CD45 incluster permL	0.8569066	0.1127983

cCTC Longitudinal Analysis by liver mets - full model

**Biomarker**	**Liver metastasis**	**Time.Effect**	**Interaction**
cCTCs permL	0.9242847	0.482884	0.3095155
CK permL	0.9211424	0.7624566	0.6374692
EpCAM permL	0.4883123	0.2545433	0.1803765
Clusters permL	0.4643683	0.0481953	0.1277263
Clustersof2permL	0.8852456	0.1693103	0.4346423
cCTCincluster permL	0.8101517	0.7809501	0.2358947
Clusters>2 permL	0.3936024	0.021446	0.1165961
CD45 incluster permL	0.293977	0.1462995	0.1428946

cCTC Longitudinal Analysis by liver mets - reduced model

Biomarker	Liver metastasis	Time Effect
cCTCs permL	0.1815107	0.7780365
CK permL	0.4428384	0.4830704
EpCAM permL	0.5781828	0.7281566
Clusters permL	0.4889495	0.1588951
Clustersof2permL	0.4280717	0.2419272
cCTCincluster permL	0.3014084	0.596671
**Clusters>2 permL**	0.5143595	**0.0862131**
CD45 incluster permL	0.7274505	0.1085877

Table S2. Full and Reduced model results for T2CTC analysis by receptor status and metastatic site

T2CTC Longitudinal Analysis by receptor status - full model

Biomarker	Receptor Effect	Time.Effect	Interaction
cCTCs permL	0.2836593	0.4482051	0.6694837
CK/Ep/Trop2 permL	0.4802839	0.8340946	0.8919177
CK/EpCAM permL	0.5649867	0.0955775	0.1789835
Trop2 Only permL	0.5484958	0.8870074	0.490742
clusters permL	0.966292	0.2493243	0.5708911
CTC in cluster permL	0.7295486	0.5810308	0.8777693
CD45 in cluster permL	0.9998403	0.2495356	0.5364823
clusters of 2 permL	0.2829963	0.6461081	0.4631998
clusters >2 permL	0.633026	0.2716701	0.4975725

T2CTC Longitudinal Analysis by receptor status - reduced model

Biomarker	Receptor Effect	Time.Effect
cCTCs permL	0.2015368	0.4379249
CK/Ep/Trop2 permL	0.3436705	0.8177385
CK/EpCAM permL	0.4330202	0.1147716
Trop2 Only permL	0.6926127	0.7719451
clusters permL	0.5605467	0.3177116
CTC in cluster permL	0.3723595	0.4720152
CD45 in cluster permL	0.5724358	0.3317939
clusters of 2 permL	0.3670707	0.91639
clusters >2 permL	0.578408	0.2320216

T2CTC Longitudinal Analysis by lung mets - full model

Biomarker	Lung metastasis	Time Effect	Interaction
cCTCs permL	0.9063863	0.6646335	0.3337083
CK/Ep/Trop2 permL	0.7274902	0.310993	0.9008095
CK/EpCAM permL	0.7055956	0.1634125	0.4115009
Trop2 Only permL	0.5808711	0.6954675	0.5588975
clusters permL	0.2043378	0.0990788	0.5698739
CTC in cluster permL	0.337467	0.1632171	0.6860191
CD45 in cluster permL	0.2114308	0.106089	0.5954148
clusters of 2 permL	0.8539235	0.4408509	0.5483344
clusters >2 permL	0.7970913	0.1349175	0.9024301

T2CTC Longitudinal Analysis by lung mets - reduced model

Biomarker	Lung metastasis	Time.Effect
cCTCs permL	0.3022704	0.8887667
CK/Ep/Trop2 permL	0.7225444	0.2983159
CK/EpCAM permL	0.623703	0.1208642
Trop2 Only permL	0.9967088	0.67912
**clusters permL**	0.2517078	**0.0850304**
CTC in cluster permL	0.3649019	0.1518455
CD45 in cluster permL	0.2475776	0.103944
clusters of 2 permL	0.1784606	0.6210561
clusters >2 permL	0.4802524	0.1188084

T2CTC Longitudinal Analysis by bone mets - full model

Biomarker	Bone metastasis	Time Effect	Interaction
cCTCs permL	0.1024883	0.7607956	0.4544681
CK/Ep/Trop2 permL	0.5332772	0.8543216	0.8250762
CK/EpCAM permL	0.9993766	0.1934376	0.9399574
**Trop2 Only permL**	**0.0425536**	0.1940793	**0.0516383**
clusters permL	0.3030117	0.215345	0.2858936
CTC in cluster permL	0.7889192	0.4010738	0.2148713
CD45 in cluster permL	0.2455702	0.3227855	0.3853668
clusters of 2 permL	0.9597619	0.9433401	0.7847325
clusters >2 permL	0.3810536	0.6627255	0.3652125

T2CTC Longitudinal Analysis by bone mets - reduced model

Biomarker	Bone metastasis	Time.Effect
cCTCs permL	0.1058522	0.6207421
CK/Ep/Trop2 permL	0.3345792	0.8296573
CK/EpCAM permL	0.961045	0.1770317
Trop2 Only permL	0.1558367	0.9669642
clusters permL	0.8103949	0.172671
CTC in cluster permL	0.5291456	0.3909038
CD45 in cluster permL	0.7869444	0.1871328
clusters of 2 permL	0.5733835	0.7540823
clusters >2 permL	0.9849838	0.1334312

T2CTC Longitudinal Analysis by brain mets - full model

Biomarker	Brain metastasis	Time.Effect	Interaction
cCTCs permL	0.2621332	0.8557758	0.1162863
CK/Ep/Trop2 permL	0.8778475	0.3111152	0.1364921
CK/EpCAM permL	0.3391488	0.2083395	0.7117655
Trop2 Only permL	0.1473407	0.9340654	0.126343
clusters permL	0.4618104	0.1149915	0.5656991
CTC in cluster permL	0.6842667	0.1336075	0.2396501
CD45 in cluster permL	0.4342613	0.1319909	0.6218055
clusters of 2 permL	0.333514	0.8439817	0.6930915
**clusters >2 permL**	**0.0088152**	0.011847	**0.0208443**

T2CTC Longitudinal Analysis by brain mets - reduced model

Biomarker	Brain metastasis	Time.Effect
cCTCs permL	0.9112352	0.7944408
CK/Ep/Trop2 permL	0.0452681	0.5639709
CK/EpCAM permL	0.2829749	0.167068
Trop2 Only permL	0.9864225	0.6748743
clusters permL	0.1937652	0.0740681
**CTC in cluster permL**	**0.0857766**	0.1412289
**CD45 in cluster permL**	0.2068656	**0.0760479**
clusters of 2 permL	0.2334044	0.6204525
clusters >2 permL	0.3707642	0.2269231

T2CTC Longitudinal Analysis by liver mets - full model

Biomarker	Liver metastasis	Time.Effect	Interaction
cCTCs permL	0.8470157	0.4909294	0.9893393
CK/Ep/Trop2 permL	0.9168901	0.3262512	0.4840867
CK/EpCAM permL	0.6453493	0.1625714	0.8412764
Trop2 Only permL	0.5408545	0.6307476	0.6307476
clusters permL	0.4416415	0.1197978	0.2060446
CTC in cluster permL	0.6716213	0.2414042	0.3589554
CD45 in cluster permL	0.4330292	0.121603	0.1945085
clusters of 2 permL	0.6919921	0.5579575	0.8224005
clusters >2 permL	0.5682477	0.2029562	0.4795684

T2CTC Longitudinal Analysis by liver mets - reduced model

Biomarker	Liver metastasis	Time.Effect
cCTCs permL	0.8587662	0.5497337
CK/Ep/Trop2 permL	0.5589497	0.4693915
CK/EpCAM permL	0.6487296	0.142409
Trop2 Only permL	0.6388642	0.6106731
clusters permL	0.7065025	0.2394939
CTC in cluster permL	0.7113897	0.2973394
CD45 in cluster permL	0.6931977	0.2596159
clusters of 2 permL	0.3227521	0.4788816
clusters >2 permL	0.9024607	0.145506

Table S3. Model parameters for cCTC analysis by receptor status and metastatic site

Biomarker	HR+		HER2+		Difference in slope	
	Intercept (95% CI)	Slope (95% CI)	Intercept (95% CI)	Slope (95% CI)	Difference (95% CI)	p.value
cCTCs	1.280 (0.647, 1.913)	0.001 (-0.014, 0.015)	1.067 (0.313, 1.822)	0.002 (-0.012, 0.017)	0.002 (-0.018, 0.022)	0.841
CK	2.367 (1.699, 3.034)	-0.002 (-0.019, 0.015)	2.024 (1.363, 2.685)	0.008 (-0.009, 0.025)	0.010 (-0.015, 0.034)	0.407
**EpCAM**	1.990 (1.290, 2.690)	-0.005 (-0.018, 0.007)	1.793 (0.955, 2.631)	0.019 (0.003, 0.035)	0.024 (0.004, 0.045)	**0.022**
Clusters	2.316 (1.671, 2.961)	0.002 (-0.011, 0.015)	2.346 (1.617, 3.074)	0.021 (0.005, 0.036)	0.019 (-0.001, 0.039)	**0.067**
Clustersof2	1.854 (1.296, 2.412)	0.001 (-0.010, 0.011)	2.028 (1.368, 2.687)	0.014 (0.001, 0.028)	0.014 (-0.003, 0.030)	0.112
cCTCincluster	0.728 (0.248, 1.208)	-0.000 (-0.011, 0.011)	0.445 (-0.114, 1.004)	0.004 (-0.007, 0.015)	0.004 (-0.011, 0.019)	0.569
**Clusters>2**	1.534 (0.988, 2.079)	0.000 (-0.010, 0.010)	1.273 (0.653, 1.894)	0.023 (0.010, 0.037)	0.023 (0.006, 0.040)	**0.007**
CD45 incluster	2.089 (1.513, 2.664)	0.002 (-0.009, 0.013)	2.111 (1.461, 2.760)	0.017 (0.003, 0.030)	0.014 (-0.003, 0.032)	0.109

Biomarker	No Bone Metastasis		Bone Metastasis		Difference in slope	
	Intercept (95% CI)	Slope (95% CI)	Intercept (95% CI)	Slope (95% CI)	Difference (95% CI)	p.value
cCTCs	1.073 (0.095, 2.050)	-0.006 (-0.027, 0.014)	1.488 (0.874, 2.103)	-0.001 (-0.011, 0.009)	0.006 (-0.017, 0.028)	0.608
CK	2.296 (1.618, 2.974)	0.000 (-0.021, 0.022)	2.065 (1.474, 2.656)	0.005 (-0.009, 0.020)	0.005 (-0.020, 0.030)	0.692
EpCAM	2.132 (0.810, 3.453)	0.009 (-0.025, 0.042)	1.966 (1.101, 2.832)	0.000 (-0.017, 0.018)	-0.008 (-0.046, 0.030)	0.630
Clusters	2.579 (1.789, 3.368)	0.011 (-0.013, 0.036)	2.225 (1.584, 2.866)	0.009 (-0.006, 0.024)	-0.002 (-0.030, 0.027)	0.895
Clustersof2	2.062 (1.376, 2.749)	0.009 (-0.012, 0.030)	1.840 (1.290, 2.391)	0.006 (-0.007, 0.018)	-0.003 (-0.028, 0.021)	0.770
cCTCincluster	0.468 (-0.546, 1.481)	-0.003 (-0.018, 0.013)	1.130 (0.514, 1.746)	-0.005 (-0.014, 0.004)	-0.003 (-0.020, 0.015)	0.767
Clusters>2	1.541 (0.594, 2.488)	0.014 (-0.013, 0.041)	1.400 (0.754, 2.046)	0.009 (-0.006, 0.024)	-0.005 (-0.036, 0.025)	0.716
CD45 incluster	2.074 (1.373, 2.774)	0.015 (-0.005, 0.036)	2.038 (1.480, 2.596)	0.007 (-0.006, 0.019)	-0.009 (-0.032, 0.015)	0.463

Biomarker	No Brain Metastasis		Brain Metastasis		Difference in slope	
	Intercept (95% CI)	Slope (95% CI)	Intercept (95% CI)	Slope (95% CI)	Difference (95% CI)	p.value
cCTCs	1.195 (0.550, 1.840)	-0.001 (-0.014, 0.013)	1.550 (0.648, 2.452)	0.003 (-0.011, 0.016)	0.003 (-0.014, 0.021)	0.717
CK	2.210 (1.612, 2.809)	-0.000 (-0.014, 0.013)	2.074 (1.387, 2.761)	0.017 (0.001, 0.033)	0.017 (-0.004, 0.038)	0.106
EpCAM	1.980 (1.184, 2.775)	0.003 (-0.015, 0.021)	1.943 (0.821, 3.066)	0.001 (-0.024, 0.027)	-0.002 (-0.032, 0.029)	0.913
Clusters	2.387 (1.721, 3.054)	0.005 (-0.010, 0.021)	2.401 (1.647, 3.154)	0.015 (-0.004, 0.034)	0.010 (-0.015, 0.035)	0.403
Clustersof2	1.999 (1.401, 2.596)	0.001 (-0.012, 0.014)	1.918 (1.048, 2.789)	0.014 (-0.004, 0.032)	0.013 (-0.009, 0.035)	0.214
cCTCincluster	0.830 (0.206, 1.455)	-0.004 (-0.012, 0.004)	0.900 (0.011, 1.789)	0.003 (-0.012, 0.019)	0.008 (-0.010, 0.025)	0.363
Clusters>2	1.401 (0.801, 2.000)	0.009 (-0.007, 0.024)	1.465 (0.775, 2.154)	0.013 (-0.006, 0.032)	0.005 (-0.020, 0.029)	0.699
CD45 incluster	2.149 (1.561, 2.736)	0.005 (-0.008, 0.019)	1.958 (1.293, 2.623)	0.013 (-0.003, 0.030)	0.008 (-0.014, 0.029)	0.449

Biomarker	No Liver Metastasis		Liver Metastasis		Difference in slope	
	Intercept (95% CI)	Slope (95% CI)	Intercept (95% CI)	Slope (95% CI)	Difference (95% CI)	p.value
cCTCs	1.186 (0.442, 1.930)	-0.002 (-0.011, 0.008)	1.235 (0.469, 2.002)	0.008 (-0.008, 0.025)	0.010 (-0.009, 0.029)	0.310
CK	2.247 (1.673, 2.821)	0.005 (-0.008, 0.018)	2.199 (1.429, 2.969)	-0.001 (-0.024, 0.022)	-0.006 (-0.032, 0.020)	0.637
EpCAM	1.990 (1.154, 2.826)	-0.002 (-0.019, 0.015)	1.569 (0.623, 2.515)	0.018 (-0.008, 0.044)	0.020 (-0.011, 0.050)	0.180
Clusters	2.459 (1.704, 3.214)	0.003 (-0.009, 0.015)	2.039 (1.171, 2.907)	0.022 (0.000, 0.043)	0.019 (-0.006, 0.043)	0.128
Clustersof2	1.893 (1.237, 2.550)	0.003 (-0.008, 0.015)	1.822 (1.061, 2.584)	0.012 (-0.008, 0.032)	0.009 (-0.014, 0.031)	0.435
cCTCincluster	0.791 (0.049, 1.533)	-0.004 (-0.012, 0.005)	0.677 (-0.043, 1.397)	0.006 (-0.008, 0.020)	0.010 (-0.007, 0.026)	0.236
Clusters>2	1.479 (0.903, 2.056)	0.005 (-0.007, 0.017)	1.074 (0.330, 1.817)	0.024 (0.003, 0.044)	0.019 (-0.005, 0.043)	0.117
CD45 incluster	2.474 (1.444, 3.504)	-0.000 (-0.013, 0.013)	1.746 (0.706, 2.786)	0.018 (-0.003, 0.039)	0.018 (-0.007, 0.043)	0.143

Biomarker	No Lung Metastasis		Lung Metastasis		Difference in slope	
	Intercept (95% CI)	Slope (95% CI)	Intercept (95% CI)	Slope (95% CI)	Difference (95% CI)	p.value
cCTCs	1.633 (0.899, 2.366)	-0.001 (-0.016, 0.014)	0.985 (0.318, 1.652)	0.003 (-0.010, 0.015)	0.003 (-0.016, 0.022)	0.715
CK	2.285 (1.664, 2.905)	0.011 (-0.005, 0.027)	1.959 (1.363, 2.555)	0.003 (-0.012, 0.017)	-0.009 (-0.030, 0.013)	0.407
EpCAM	2.364 (1.323, 3.404)	-0.004 (-0.029, 0.021)	1.673 (0.859, 2.487)	0.007 (-0.011, 0.025)	0.011 (-0.020, 0.041)	0.465
Clusters	2.692 (1.947, 3.437)	0.008 (-0.013, 0.029)	2.077 (1.405, 2.749)	0.010 (-0.006, 0.026)	0.002 (-0.024, 0.028)	0.859
Clustersof2	2.083 (1.447, 2.719)	0.008 (-0.009, 0.025)	1.780 (1.202, 2.358)	0.005 (-0.008, 0.018)	-0.003 (-0.024, 0.018)	0.776
cCTCincluster	0.986 (0.113, 1.860)	-0.001 (-0.018, 0.016)	0.801 (0.108, 1.493)	-0.003 (-0.012, 0.006)	-0.002 (-0.021, 0.017)	0.820
Clusters>2	1.704 (1.014, 2.395)	0.009 (-0.011, 0.028)	1.173 (0.570, 1.777)	0.012 (-0.003, 0.027)	0.003 (-0.022, 0.027)	0.810
CD45 incluster	2.302 (1.662, 2.941)	0.009 (-0.008, 0.026)	1.808 (1.220, 2.397)	0.010 (-0.004, 0.024)	0.001 (-0.021, 0.023)	0.942

Table S4. Model parameters for for T2CTC analysis by receptor status and metastatic site

**Biomarker**	**HR+**		**HER2+**		**Difference in slope**	
	Intercept (95% CI)	Slope (95% CI)	Intercept (95% CI)	Slope (95% CI)	Difference (95% CI)	p.value
cCTCs	2.692 (1.036, 4.349)	-0.021 (-0.058, 0.017)	1.367 (-1.293, 4.027)	-0.006 (-0.075, 0.063)	0.015 (-0.059, 0.088)	0.669
CK/Ep/Trop2	1.060 (-0.586, 2.706)	0.001 (-0.042, 0.045)	0.213 (-3.138, 3.563)	0.006 (-0.063, 0.074)	0.005 (-0.064, 0.073)	0.892
CK/EpCAM	0.515 (-0.036, 1.065)	0.002 (-0.014, 0.018)	0.310 (-0.133, 0.752)	0.018 (0.001, 0.035)	0.016 (-0.008, 0.040)	0.179
Trop2 Only	0.142 (-0.262, 0.546)	-0.005 (-0.020, 0.010)	-0.038 (-0.516, 0.441)	0.003 (-0.016, 0.022)	0.008 (-0.016, 0.032)	0.491
clusters	0.415 (-1.081, 1.912)	0.024 (-0.037, 0.085)	0.378 (-4.200, 4.957)	0.008 (-0.094, 0.111)	-0.016 (-0.072, 0.040)	0.571
CTC in cluster	0.535 (-1.608, 2.677)	0.014 (-0.049, 0.076)	.032 (-576.207, 576.270	0.008 (-0.291, 0.307)	-0.006 (-0.166, 0.154)	0.878
CD45 in cluster	0.383 (-1.110, 1.877)	0.025 (-0.033, 0.082)	0.383 (-1.325, 2.091)	0.008 (-0.041, 0.056)	-0.017 (-0.073, 0.039)	0.536
clusters of 2	1.128 (-0.341, 2.596)	-0.005 (-0.045, 0.034)	-0.190 (-2.293, 1.913)	0.023 (-0.046, 0.092)	0.028 (-0.049, 0.106)	0.463
clusters >2	-0.397 (-1.461, 0.666)	0.043 (-0.018, 0.103)	-0.012 (-1.223, 1.198)	0.011 (-0.074, 0.095)	-0.032 (-0.136, 0.072)	0.498

**Biomarker**	**No Bone Metastasis**		**Bone Metastasis**		**Difference in slope**	
	Intercept (95% CI)	Slope (95% CI)	Intercept (95% CI)	Slope (95% CI)	Difference (95% CI)	p.value
cCTCs	0.943 (-1.717, 3.604)	0.008 (-0.121, 0.137)	2.526 (1.209, 3.843)	-0.018 (-0.063, 0.027)	-0.026 (-0.105, 0.053)	0.454
CK/Ep/Trop2	0.206 (-13.699, 14.111)	-0.001 (-0.478, 0.477)	0.819 (-0.495, 2.134)	0.007 (-0.044, 0.059)	0.008 (-0.101, 0.116)	0.825
CK/EpCAM	0.384 (-0.058, 0.825)	0.009 (-0.013, 0.031)	0.384 (-0.104, 0.872)	0.008 (-0.008, 0.025)	-0.001 (-0.028, 0.026)	0.940
Trop2 Only	0.481 (0.012, 0.949)	-0.019 (-0.040, 0.001)	-0.096 (-0.394, 0.202)	0.004 (-0.007, 0.015)	0.023 (-0.000, 0.046)	**0.052**
clusters	0.522 (-0.350, 1.394)	0.004 (-0.108, 0.116)	-0.195 (-1.315, 0.925)	0.042 (-0.003, 0.087)	0.038 (-0.049, 0.125)	0.286
CTC in cluster	0.145 (-0.643, 0.932)	-0.006 (-0.052, 0.040)	-0.037 (-1.162, 1.088)	0.030 (-0.014, 0.073)	0.036 (-0.026, 0.098)	0.215
CD45 in cluster	0.514 (-0.659, 1.686)	0.004 (-0.296, 0.304)	-0.300 (-1.381, 0.780)	0.047 (-0.012, 0.105)	0.043 (-0.141, 0.226)	0.385
clusters of 2	0.504 (-2.394, 3.402)	-0.011 (-0.129, 0.108)	0.577 (-0.626, 1.780)	0.006 (-0.039, 0.051)	0.017 (-0.105, 0.138)	0.785
clusters >2	0.422 (-1.375, 2.218)	-0.016 (-0.141, 0.110)	-0.456 (-1.321, 0.409)	0.044 (-0.011, 0.098)	0.059 (-0.076, 0.195)	0.365

**Biomarker**	**No Brain Metastasis**		**Brain Metastasis**		**Difference in slope**	
	Intercept (95% CI)	Slope (95% CI)	Intercept (95% CI)	Slope (95% CI)	Difference (95% CI)	p.value
cCTCs	2.218 (0.938, 3.499)	-0.022 (-0.075, 0.030)	1.290 (0.241, 2.339)	0.028 (-0.010, 0.065)	0.050 (-0.014, 0.114)	0.116
CK/Ep/Trop2	0.649 (-0.611, 1.909)	-0.006 (-0.045, 0.032)	0.806 (-1.676, 3.288)	0.033 (-0.009, 0.075)	0.040 (-0.013, 0.092)	0.136
CK/EpCAM	0.512 (0.035, 0.990)	0.006 (-0.014, 0.025)	0.169 (-0.455, 0.792)	0.010 (-0.012, 0.033)	0.004 (-0.024, 0.033)	0.712
Trop2 Only	0.207 (-0.101, 0.515)	-0.008 (-0.020, 0.005)	-0.206 (-0.690, 0.277)	0.008 (-0.009, 0.026)	0.016 (-0.005, 0.037)	0.126
clusters	0.306 (-0.776, 1.389)	0.013 (-0.030, 0.055)	0.832 (-0.471, 2.134)	0.027 (-0.005, 0.058)	0.014 (-0.035, 0.063)	0.566
CTC in cluster	0.213 (-0.903, 1.329)	0.004 (-0.032, 0.040)	0.566 (-1.624, 2.756)	0.032 (-0.006, 0.070)	0.028 (-0.020, 0.076)	0.240
CD45 in cluster	0.289 (-0.781, 1.359)	0.013 (-0.031, 0.057)	0.823 (-0.080, 1.727)	0.026 (-0.004, 0.055)	0.012 (-0.040, 0.064)	0.622
clusters of 2	0.117 (-0.922, 1.156)	0.014 (-0.037, 0.065)	1.473 (-1.156, 4.101)	-0.005 (-0.110, 0.101)	-0.019 (-0.136, 0.098)	0.693
clusters >2	0.055 (-0.773, 0.884)	0.010 (-0.051, 0.070)	-3.383 (-5.767, -0.999)	0.171 (0.054, 0.288)	0.161 (0.030, 0.293)	**0.021**

**Biomarker**	**No Liver Metastasis**		**Liver Metastasis**		**Difference in slope**	
	Intercept (95% CI)	Slope (95% CI)	Intercept (95% CI)	Slope (95% CI)	Difference (95% CI)	p.value
cCTCs	1.544 (0.655, 2.434)	0.012 (-0.038, 0.063)	1.366 (-0.254, 2.987)	0.012 (-0.054, 0.079)	-0.000 (-0.082, 0.081)	0.989
CK/Ep/Trop2	0.404 (-0.566, 1.375)	0.004 (-0.026, 0.035)	0.316 (-1.170, 1.801)	0.026 (-0.029, 0.080)	0.021 (-0.040, 0.083)	0.484
CK/EpCAM	0.439 (-0.001, 0.880)	0.009 (-0.013, 0.030)	0.266 (-0.368, 0.901)	0.011 (-0.011, 0.034)	0.003 (-0.027, 0.033)	0.841
Trop2 Only	0.000 (-0.390, 0.390)	-0.000 (-0.016, 0.016)	0.158 (-0.214, 0.531)	-0.005 (-0.019, 0.009)	-0.005 (-0.027, 0.017)	0.631
clusters	0.405 (-0.371, 1.181)	0.005 (-0.053, 0.063)	-0.192 (-1.540, 1.157)	0.047 (-0.006, 0.100)	0.041 (-0.027, 0.110)	0.206
CTC in cluster	0.177 (-0.597, 0.951)	0.005 (-0.086, 0.095)	-0.142 (-1.456, 1.171)	0.034 (-0.019, 0.088)	0.030 (-0.044, 0.103)	0.359
CD45 in cluster	0.404 (-0.371, 1.180)	0.005 (-0.054, 0.063)	-0.203 (-1.548, 1.143)	0.047 (-0.006, 0.100)	0.043 (-0.026, 0.112)	0.195
clusters of 2	0.065 (-1.458, 1.588)	0.007 (-0.071, 0.085)	0.450 (-0.894, 1.793)	0.016 (-0.044, 0.075)	0.009 (-0.088, 0.105)	0.822
clusters >2	-0.045 (-1.133, 1.043)	0.014 (-0.071, 0.100)	-0.488 (-1.586, 0.610)	0.047 (-0.013, 0.106)	0.032 (-0.071, 0.136)	0.480

**Biomarker**	**No Lung Metastasis**		**Lung Metastasis**		**Difference in slope**	
	Intercept (95% CI)	Slope (95% CI)	Intercept (95% CI)	Slope (95% CI)	Difference (95% CI)	p.value
cCTCs	1.562 (0.642, 2.481)	0.021 (-0.011, 0.054)	1.661 (0.187, 3.135)	-0.008 (-0.070, 0.053)	-0.030 (-0.096, 0.036)	0.334
CK/Ep/Trop2	0.586 (-0.957, 2.128)	0.013 (-0.024, 0.050)	0.288 (-1.179, 1.756)	0.017 (-0.040, 0.073)	0.004 (-0.055, 0.062)	0.901
CK/EpCAM	0.342 (-0.065, 0.749)	0.013 (-0.002, 0.029)	0.470 (-0.076, 1.016)	0.004 (-0.016, 0.023)	-0.010 (-0.034, 0.015)	0.412
Trop2 Only	0.141 (-0.239, 0.521)	-0.005 (-0.020, 0.010)	-0.003 (-0.388, 0.381)	0.001 (-0.014, 0.016)	0.006 (-0.015, 0.027)	0.559
clusters	0.680 (-0.064, 1.424)	0.018 (-0.022, 0.058)	-0.272 (-1.580, 1.036)	0.035 (-0.019, 0.089)	0.017 (-0.048, 0.082)	0.570
CTC in cluster	0.488 (-0.399, 1.375)	0.014 (-0.014, 0.041)	-0.229 (-1.528, 1.070)	0.025 (-0.029, 0.078)	0.011 (-0.046, 0.067)	0.686
CD45 in cluster	0.678 (-0.072, 1.428)	0.018 (-0.024, 0.060)	-0.259 (-1.566, 1.048)	0.034 (-0.020, 0.088)	0.016 (-0.050, 0.081)	0.595
clusters of 2	0.451 (-1.249, 2.151)	0.023 (-0.032, 0.079)	0.263 (-1.027, 1.553)	0.003 (-0.066, 0.072)	-0.020 (-0.097, 0.056)	0.548
clusters >2	-0.190 (-1.337, 0.958)	0.038 (-0.037, 0.113)	-0.388 (-1.405, 0.628)	0.033 (-0.034, 0.100)	-0.005 (-0.106, 0.095)	0.902
